# Transcatheter Treatment of Bicuspid Aortic Valve Disease: Imaging and Interventional Considerations

**DOI:** 10.3389/fcvm.2018.00091

**Published:** 2018-07-19

**Authors:** Rajiv Das, Rishi Puri

**Affiliations:** ^1^Freeman Hospital, Newcastle upon Tyne, United Kingdom; ^2^Department of Cardiovascular Medicine, Cleveland Clinic, Cleveland, OH, United States

**Keywords:** TAVI, bicuspid valve disease, CT, treatment, bicuspid aortic valve

## Abstract

Patients with bicuspid aortic valve disease have systematically been excluded from large randomized clinical trials investigating transcatheter aortic valve implantation (TAVI) due to their younger age, lower surgical risk and complex aortic anatomy. The asymmetric nature of the bicuspid valve orifice often accompanied by heavy regional calcification has led to concerns regarding valve positioning and expansion. Bicuspid aortic valve disease patients are at heightened risk of TAVI-related complications including coronary occlusion, aortic dissection and annular rupture, as well as the known risks of progressive aortopathy in these patients. These unique anatomical characteristics pose challenges for TAVI operators. However, with recent and ongoing refinements in implantation technique, improvements in pre-procedural imaging and iterations in device design, TAVI is emerging as a safe and feasible treatment option in this population. Paravalvular aortic regurgitation and high pacemaker rates have been the Achilles Heel for TAVI in bicuspid valve patients, yet newer generation devices are yielding promising results. Further studies are required before TAVI ultimately emerges as a viable option in low and intermediate surgical-risk patients with bicuspid valve disease. This review comprehensively summarizes the epidemiology, pathology and current evidence for TAVI in patients with bicuspid aortic valve disease. We also outline some practical tips for performing TAVI in these patients.

## Introduction

The transcatheter aortic valve implantation (TAVI) revolution for severe tricuspid aortic valve stenosis (AS) is well-recognized as an alternative to surgical aortic valve replacement (SAVR) for severe aortic stenosis. This has been established in the randomized clinical trials for balloon-expandable and self-expanding valves (1–4). TAVI is now regarded as the standard of care for patients with severe symptomatic AS that are considered inoperable or in patients at high surgical risk. More recently randomized clinical trials have shown non-inferiority when TAVI has been compared with SAVR in patients at intermediate or low surgical risk (5–7). Bicuspid aortic valve (BAV) has largely been excluded from seminal randomized clinical trials involving TAVI. This was due to concerns about (i) valve positioning and expansion due to the asymmetrical nature of the leaflets and heavy calcification leading to severe paravalvular leak (PVL), (ii) aortic annulus rupture and risk of coronary occlusion, (iii) concomitant aortopathy associated with BAV increasing the risk of spontaneous and iatrogenic aortic dissection and rupture, and (iv) concerns regarding the long-term durability of Transcatheter Heart Valves (THV), particularly in a younger BAV population. There is clear data on the safety and efficacy of TAVI in patients with tricuspid valve severe AS (1–10), and despite encouraging data from registries including BAV disease patients (11–14), TAVI has yet to establish itself in this patient cohort.

This review summarizes the evidence for TAVI in bicuspid aortic valve disease, the role of multi-slice computed tomography (MSCT) to aid procedural planning, and technical considerations to undertake when performing TAVI in BAV.

## Epidemiology

BAV disease is a common congenital cardiac abnormality seen in adults and is frequently associated with AS (15). The estimated incidence of bicuspid aortic valves is 0.4–2.25% in the general population. The most frequent complication of BAV is AS, often requiring aortic valve replacement surgery. BAV is commonly associated with aortopathy leading to asymptomatic dilatation of the ascending aorta in the initial stages followed by aneurysm formation of the aorta and the potential life-threatening complication of aortic dissection (16–20). In a large population based study involving nearly 200 patients with a mean follow-up of 15 years, 13% developed severe AS requiring SAVR. In this cohort of patients, the main indication for surgery was severe AS; performed at a younger age group compared with the general population (21).

Registry data has shown that 37% of BAV patients have moderate-to-severe AS at the time of their initial echocardiogram (22). The prevalence of bicuspid aortic valve disease in patients undergoing surgical aortic valve replacement has been reported to be as high as 50% in some surgical series, 27.5% amongst octogenarians, and up to 41.7% of septuagenarians (23). In an Asian transcatheter aortic valve implantation (TAVI) population, BAV has been reported in upto 50%, potentially posing unique challenges for percutaneous treatment options in the Asian landscape (24).

## Pathology

BAV disease is frequently associated with valvular stenosis, valvular regurgitation, aortic coarctation, aortic dilatation, aneurysms, and dissection. It is essential that pre-procedural imaging assesses the thoracic aorta in BAV patients. Aortic root dilatation occurs in 50–60% of patients with a normally functioning bicuspid valve, increasing the risk of aortic dissection nine-fold (25). The etiology of ascending aortic dilatation may be due to genetic and hemodynamic factors that affect the aortic wall elasticity and strength. Genetic mutations in smooth muscle cells α actin (ACTA2) and transforming growth factor β receptor (TGFBR1 and TGFBR2) have been linked with aortopathy in BAV disease (26). A genetic link between aortic dilatation and BAV can be substantiated by the fact there is greater incidence of aortopathy in first degree relatives with BAV disease, and not infrequently we see progressive aortic root dilatation in patients post SAVR with BAV disease (27).

**Figure 1:**
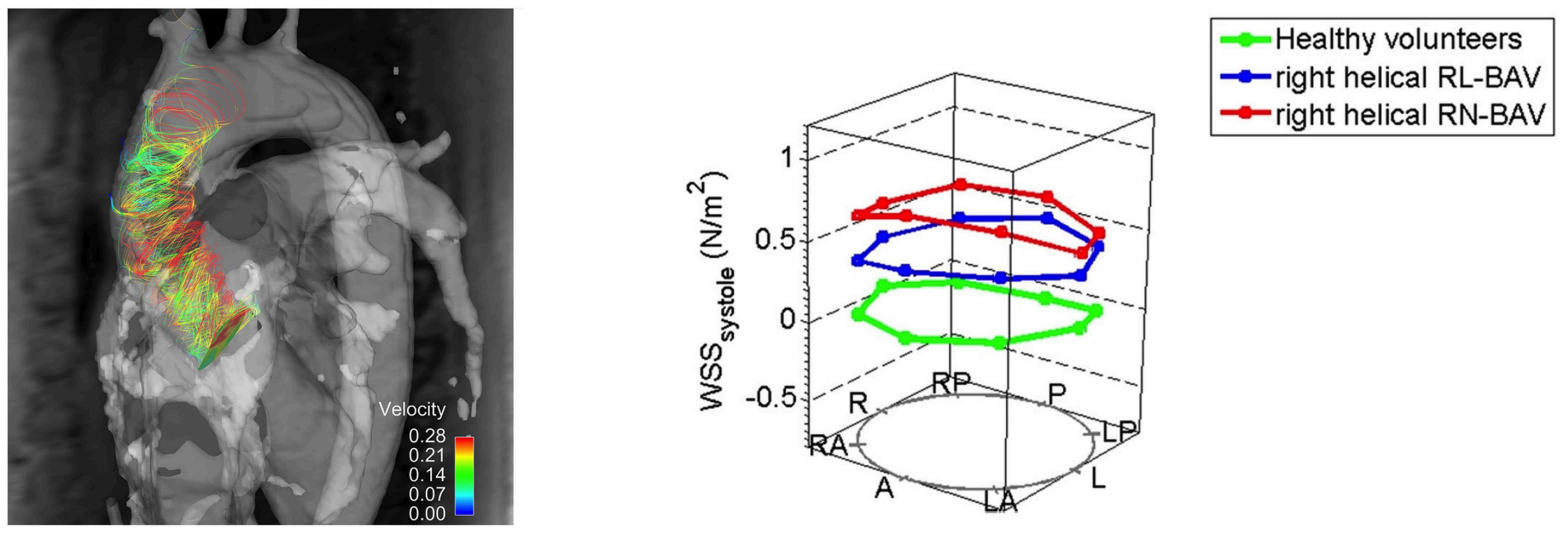
Altered right-handed helical aortic flow patterns in patients with bicuspid aortic valve disease. Patients with right and non-coronary cusp fusion BAV had higher wall shear stress patterns in the ascending aorta [Reproduced with permission from the Bissell et al. (32)].

Abnormalities in wall shear stress can arise due to the asymmetrical nature of the orifice in BAV patients. Studies using flow-sensitive MRI and four-dimensional (4D) cardiovascular MRI have looked at abnormalities in wall shear stress and flow patterns in the aortic wall for different BAV fusion patterns, which in turn has been linked with adverse remodeling within the aortic root wall (28, 29). The right and left cusp fusion variant of BAV is associated with asymmetrically elevated wall shear stress in the ascending aorta (30, 31). Phenotypic variations in BAV fusion patterns may need to be considered when assessing patients, especially if TAVI is to be extended to low-risk patients. As highlighted certain BAV fusion patterns are predictors of increased wall shear stress and aortic root dilatation (Figure 1).

## Classification systems for bicuspid aortic valve disease

**Figure 2:**
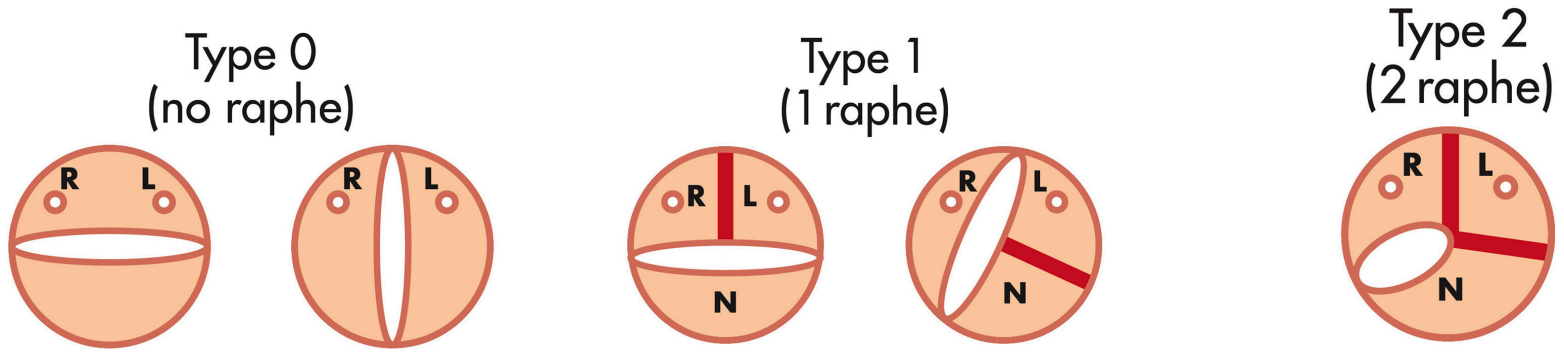
The Sievers and Schmidtke classification system for bicuspid aortic valve [Adapted from Sievers et al. (33)].

Different classification systems exist for the varying BAV morphologies; based on the presence and characteristics of the raphe, commissural position, description of the cusp and its size and the aortic sinus characteristics (15, 21). The most widely used classification is by Sievers and Schmidtke; due to its simplicity and user friendliness (33). Valve morphology is classified according to the number of cusps and the presence of raphes, as well as the position and symmetry of the cusps. Type 0 has 2 symmetric leaflet/cusps and 1 commissure without evidence of a raphe, Type 1 has a single raphe due to fusion of the left coronary cusp with either the right or non-coronary cusp, and Type 2 arises when 2 raphes are present with fusion of both the right and non-coronary cusps (Figure 2). A functional (bicuspid) valve are classified as tricuspid valves with no raphe present, but there is fixation of the commissure between 2 cusps due to degenerative processes. Mylotte et al. used a modified Sievers classification system and observed higher rates of PVL in Sievers type 1 morphology (34.2%) than in Sievers type 0 (13.3%), possibly due to incomplete THV expansion due to calcified raphe and leaflet asymmetry (14).

Jilaihawi et al. proposed a classification system for BAV and described 3 subtypes, tricommissural, bicommisural raphe type, and bicommisural non-raphe type (11). The classification enabled a greater understanding of the interaction of the valve with the aortic-valvular complex at both the basal leaflet plane (presence or absence of a raphe) and at the commissural level (presence of 2 or 3 commissures). It was noted that the presence of a calcified raphe may impact on TAVI expansion and device apposition at the annulus. Tricommisural BAV type was not found to be associated with aortopathy and has widely been termed functional or acquired BAV disease. Tricommisural BAV arises from rheumatic, fibrotic, or calcific processes leading to focal commissural fusion (11). Tricommisural BAV disease is different from tricuspid valve aortic stenosis and may account for the higher incidence of PPM after TAVI. Interestingly, there were marked geographical differences between the subtypes of BAV. Non-raphe type bicommisural bicuspid AS was more common in Asia, but not in North America or Europe. The classification system did not predict the rates of moderate or severe PVL (tricommisural 19%, bicommisural raphe type 19.5%, and bicommisural non-raphe subtype 15%). Thirty-day mortality rates according to the BAV subtypes were not found to be statistically different (tricommisural 4.2%, bicommissural raphe subtype 2.7%, and bicommisural non-raphe subtype 9.5%). There was also no difference in new permanent pacemaker implantation rates between the BAV subtypes.

## Current registry data on bicuspid aortic valve disease

Our current understanding of the safety and efficacy of treating BAV patients with TAVI is largely based on small registries, most of which used older generation THVs (14, 34, 35). A multi-center study raised concerns regarding an excess of bioprosthetic PVL in bicuspid aortic valve disease patients undergoing TAVI (14). This was a retrospective registry of 139 patients across 12 centers collecting clinical, procedural, and follow-up data. Procedural mortality was 3.6%, with THV embolization occurring in 2.2% with a 1-year mortality of 17.5%. MSCT-based TAVI sizing was used in 63.5% of patients. AR grade 2+ post-TAVI was not infrequent at 28.4% which decreased to 17.4% when CT-sizing and planning algorithms were used. This series demonstrated that pre-procedural MSCT imaging can minimize PVL in TAVI for BAV disease by more accurately sizing the annulus. In a registry using a newer generation SAPIEN 3 valve, 51 patients with BAV disease from 8 centers were evaluated (13). The incidence of trivial and no AR post-TAVI was 63% and mild AR was 37%. The 30-day mortality rate was reported at 3.9%.

In a study of 130 patients with severe AS and BAV from 14 centers undergoing TAVI (11), the 30-day outcomes were comparable with those reported in patients with tricuspid valve stenosis (1, 2, 7, 36, 37). There was however an excess of new pacemaker implantation which was similar for balloon expandable and self-expanding valves (Table 1). An increase in significant PVL was not observed in this study as compared with tricuspid valve stenosis patients undergoing TAVI. Once again, PVL rates were lower in this study if MSCT data was used for sizing.

**Table 1:** Summary of 30-day outcomes in the main TAVI trials in patients with bicuspid aortic stenosis.

**Study**	**Patients (n)**	**BE (%)**	**SE (%)**	ME* (%)	**Death (%)**	**All CVE (%)**	**Valve embolization (%)**	**PVL > Mild (%)**	**New PPM (%)**	**Conversion to surgery (%)**	**Need for second valve (%)**	**References**
Yoon et al.	546	58	34	8	3.7	2.9	NA	10.4	15.4	2.0	4.8	(12)
Perlman et al.	51	100	0	0	3.9	1.9	0	0	23.5	0	0	(13)
Jilaihawi et al.	130	54	46	0	1.5	3.2	1.5	18.1	26.2	3.1	3.1	(11)
Mylotte et al.	139	34	66	0	5.0	2.2	2.2	28.4	23.2	2.2	3.6	(14)

ME*, mechanical expanding valve LOTUS valve; NA, not available; CVE, cerebrovascular event, stroke, or transient ischemic attack.

The Bicuspid AS TAVI multicentre registry is the largest study to date evaluating 546 patients with either bicuspid or tricuspid AS who were propensity scored matched (12). Patients were recruited from Europe, North America and the Asia specific region. The patients with bicuspid severe AS had lower STS scores and represented an intermediate risk profile population, and furthermore the use of a large prostheses was more commonly associated with bicuspid AS patients. The major findings of this study were bicuspid severe AS patients had more frequent conversion to open surgery and a significantly lower device success rate as compared with propensity matched tricuspid AS patients. There were no significant between-group differences in procedural complications such aortic root injury and moderate-to-severe PVL when new generation devices were used. All-cause mortality at 2-year follow-up was comparable between the bicuspid and tricuspid groups.

## Prosthesis choice in bicuspid valve disease

Operators need to be cognisant of the potential advantages and disadvantages of balloon-expandable and self-expanding devices in the BAV space. Balloon-expandable valves exert greater radial force as compared with self-expanding devices and may circularize the native annulus minimizing potential sites for paravalvular leaks. Mylotte et al. (14) reported outcomes on both balloon-expandable and self-expanding devices observing a greater incidence of PVL ≥2 with self-expanding valves (19.6% with Sapien XT and 32.2% with CoreValve). This may be attributable to the reduced radial strength in self-expanding devices increasing the likelihood of residual PVL. Conversely, when comparisons are made using newer generation balloon-expandable and self-expanding devices which feature an external sealing skirt there were no significant differences between the two general valve designs (38).

Rates of annular rupture have been reported to be as high as 5.3% in some series using the balloon-expandable Sapien XT valve (38). This may have been largely driven by a degree of oversizing required for device anchoring to prevent significant PVL. With improvements in design of the newer-generation balloon expandable valves there is sufficient anchoring with less oversizing which has led to acceptable rates of PVL and annular rupture (39, 40). When sizing is guided by CT annular measurements a degree of oversizing (7–13% for the S3 THV design) appears to be safe with newer generation valves, leading to a reduction in AR in patients with bicuspid valve disease.

In a series of 51 patients who underwent TAVI in bicuspid AS using the new generation balloon expandable S3 valve, device success rate was reported to be 98% with no cases of moderate to severe aortic regurgitation (13). Improvements in design of the newer generation S3 THVs have resulted in a lower profile device with more accurate positioning, and improved sealing with its polyethylene terephthalate outer skirt. The Lotus mechanical expanding valve has an outer adaptive seal with the ability to be repositioned and retrieved. Promising results were also demonstrated with regards device success when this device was used to treat bicuspid AS patients (38).

## New permanent pacemaker implantation rates for tavi in bicuspid valve disease

One of the main limitations of TAVI in tricuspid valve severe AS compared with SAVR is the high incidence of conduction abnormalities. New permanent pacemaker implantation has emerged to be an important short-term complication; reported to be around 6.0% for balloon expandable valves and up to 28.0% for self-expanding valves (41, 42). There are no specific design advances that have been incorporated in the newer generation THVs to reduce the risk of permanent pacemaker implantation. Moreover, there has been a reported increase in conduction abnormalities with newer generation devices (43). Mauri et al. identified technical and anatomical factors predisposing to new permanent pacemaker implantation (44). In this study using the new generation SAPIEN 3 THV, 33 of 229 patients received a pacemaker following TAVI. Pre-existing RBBB, left ventricular outflow tract calcification and an implantation depth defined as >25.5% of the stent frame below the annulus were each found to be important predictors of new permanent pacemaker implantation. This study highlighted important technical factors such as reducing the depth of the valve implant by a mere 3 mm reduced the need for permanent pacemaker implantation by 52%. TAVI operators will need to consider such technical aspects to reduce pacing rates, yet balance these with the risk of THV embolization with higher implants (45).

For bicuspid valve severe AS, pacemaker implantation rates were similar for balloon expandable (BE) and self-expanding (SE) THVs (11, 14). Jilaihawi et al. reported pacing rates of 25.5% for balloon expanding valves and 26.9% for self-expanding valves (11). Mylotte et al. also reported higher than expected pacing rates (16.7% for balloon expanding and 26.7% for self-expanding valves) in TAVI patients (14).

It has been postulated that the higher incidence of PPM implantation rates in BAV patients is related to asymmetric THV expansion due to resistant calcified raphe and leaflet fusion. There may be preferential expansion posteriorly to the non-coronary cusp which lies adjacent to the atrioventricular node. In tricuspid valve disease or incomplete raphe type BAV, there may be a more symmetrical expansion of THVs, thus diverting tissue away from the AV node (11). The presence of bulky calcification in the Sievers L-R Type 1 BAV may cause protrusion toward the membranous part of the interventricular septum leading to atrioventricular and intraventricular conduction block (46). The higher pacemaker rates may thus be associated with difficulty in valve positioning due to irregular leaflet shape and inability to achieve a coaxial position during valve deployment. This can often lead to lower implantation depths, known to be associated with higher pacing rates. Patients with BAV disease also tend to be younger, and as TAVI moves to intermediate and the low-risk, complications such as pacemaker implantations will be important. Understanding the factors contributing to new permanent pacemaker implantation need to be addressed with particular focus on implantation depth and important calcification in the left ventricular outflow tract.

## Coronary occlusion and annular rupture

The data on acute coronary occlusion during TAVI stems from isolated case reports and case series. The incidence is reported to be < 1% and is a rare yet potentially life-threatening (4, 47–52). Randomized control trials of patients with tricuspid severe AS; report an acute coronary obstruction incidence of 0.1–1.2%. Data from bicuspid TAVI registry data report a similar 0–1.5% incidence of acute coronary obstruction (11–14, 38). Certain factors may increase the risk of coronary obstruction post-TAVI such as female sex, coronary ostia height of < 10 mm, sinus of Valsalva dimensions and the presence of severe valve calcification (53). Most reported cases of coronary obstruction post-TAVI received a balloon-expandable THV.

Recent reports have arisen of the development of delayed coronary obstruction (DCO) occurring hours or days following TAVI. In a recently published international registry of 17,092 patients undergoing TAVI, the reported incidence of delayed coronary obstruction was 0.22% (54). DCO can be divided into early (0–7 days) and late (>7 days) post-TAVI. The etiology of DCO relates to a number factors such as a narrow sinuses of Valsalva, low coronary heights, excessive calcification, valve-in-valve TAVI and pharmacological factors such as antiplatelet and anticoagulation (54). Aortic root injury and annulus rupture likewise is a rare complication in BAV undergoing TAVI; reported to have an incidence ranging from 0 to 2% in the reported literature (11–14, 38).

## Comparison of outcomes between old and newer generation THVs in BAV

THV device iteration significantly addressed the shortcomings of earlier generation THVs which were limited by PVL. Significant PVL post-TAVI has been shown to correlate with increased mortality (37, 55–57). PVL rates have improved significantly with newer-generation devices for patients with tricuspid AS patients, and has also been seen when newer-generation THVs are used in BAV patients (13). In a recently reported BAV registry comparing older versus newer generation THVs in BAV, moderate or severe paravalvular leak was significantly less frequent with new-generation devices compared to early generation devices (0.0 vs. 8.5%; *p* = 0.002), which resulted in a higher device success rate (92.2 vs. 80.9%; *p* = 0.01) (38). When compared with TAVI in tricuspid valve stenosis, there were no differences in procedural related complications with new generation devices. This was true for cumulative mortality at 2 years which were similar for tricuspid and bicuspid valves with newer generation devices (12). This has also been seen in several other registries using the SAPIEN 3 valve in tricuspid AS (58–61). Improvements in PVL have largely been made by developing a poly-ethylene terephthalate sealing skirt along with more accurate positioning. With the Lotus valve, the incidence of moderate to severe PVL has been reported to be as low as 2.0%, largely due to the adaptive seal and optimal positioning due to device retrievability and repositionability (62).

## Technical considerations for TAVI in BAV

BAV disease poses many technical challenges for TAVI operators. Selection of the optimal angiographic projection and visualization of the aortic annulus can be difficult due to the asymmetric shape of the cusps and sinus of Valsalva. Calcium distribution throughout the aorto-annular complex is frequently asymmetric, along with raphe resistant to pre-dilatation and aortic root dilatation. These variations may promote poor valve expansion and thus adversely affect valve hemodynamics and durability which in turn can lead to high transvalvular gradients, PVL, device malpositioning, and higher permanent pacemaker rates post-TAVI (11, 12, 14, 63). The aortic annulus is often elliptical in shape, larger in size, and associated with a dilated and horizontal aorta, (64) further giving rise to difficulties in device positioning and expansion. The native valve leaflets can be capacious due to leaflet fusion resulting in longer leaflets increasing the risk of coronary obstruction (34, 65).

Himbert et al. reported on the use of self-expanding devices in 15 patients with BAV disease (66). The device was associated with non-circular expansion at the annular level which was less frequent when the device was implanted lower in the left ventricular outflow tract. In another series of 21 patients who had post-procedural MSCT imaging, non-circular expansion of the valve was seen more commonly with self-expanding valves due to the asymmetrical nature of the bicuspid valve orifice and resistant raphe (67). With balloon-expandable valves there have been reports of asymmetric longitudinal valve expansion, however further assessment is required for both self-expanding and balloon-expandable valves to ascertain if this ultimately affects leaflet motion and durability (13).

### Imaging for TAVI in bicuspid-aortic valve disease

Sizing of THVs can be difficult due to multiple anatomical considerations including a large and eccentric annulus, calcified raphe, horizontal aorta, complex calcification, and aortic root dilatation. Each of these variables can interplay and make TAVI implantation technically challenging. MSCT has enabled operators to have a better understanding of the anatomy of BAV disease, critical for procedural planning pre-TAVI to minimize complications (68).

**Figure 3:**
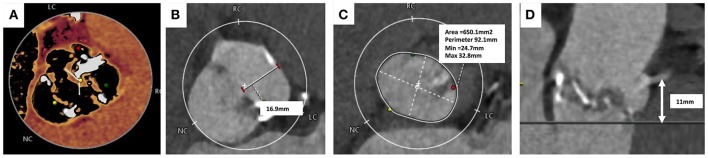
Computer Tomography Imaging of bicuspid aortic valve. **(A)** Sievers Type 1 Raphe-type bicuspid aortic valve (BAV) with mixed cusp fusion (left-right). **(B)** Large bulky cusps measuring 16.9 mm maximum diameter. **(C)** Asymmetric large annulus. **(D)** Low lying coronary ostia at 11 mm combined with large bulky leaflets indicate a risk of coronary occlusion during TAVI.

Due to the percutaneous nature of TAVI, operators lack the ability to expose the surgical field and directly visualize the aortic valve, annulus and structures around it. MSCT is used to provide a comprehensive 3-dimensional data-set of the aortic valve anatomy and identification of concomitant aortopathy. MSCT provides anatomical measurements of the aortic annulus, detail of the aortic valve, calcium burden, aortic root (“sinus of Valsalva”), coronary ostia and access site, all of which are essential to minimize complications and improve procedural outcomes. In BAV, MSCT is key to providing information on leaflet morphology, symmetry of the valve leaflets, presence of raphe and the location of calcification all of which can influence the type and size of THV selected (Figure 3) (69).

In a study using MSCT which looked at the shape and size of the annulus in bicuspid (*n* = 200) and tricuspid valves (*n* = 200), the aortic annulus was found to be less elliptical in bicuspid than tricuspid valves (ellipticity index 1.24 vs. 1.29, respectively). This study also highlighted that biscuspid valve patients had large annular areas compared with tricuspid valves (5.21 vs. 4.63 cm^2^) (27). Reports from recent large series of bicuspid patients indicate that annular dimensions still fall within the valve sizing recommendations for current commercially available THVs (13, 14, 67).

THV oversizing can lead to distortion and poor expansion of the valve prosthesis leading to PVL. This can be improved with intra-procedural post-dilatation, however there is a risk of annular rupture, aortic root haematoma and heart block with subsequent post-dilatations. A self-expanding THV may minimize the risk of annular rupture, however when compared with balloon expandable THVs, there is a greater incidence of PVL and heart block in BAV disease patients (38, 70).

### Balloon sizing

Other techniques can be used to help with valve sizing such as fluoroscopic balloon sizing of the aortic valve annulus pre-TAVI (71). Balloon sizing can complement MSCT especially when there is ambiguity regarding valve sizing and when measurements fall in the “gray zone” between two valve sizes. In the presence of bulky cusps or long leaflets, balloon sizing can mimic valve implantation and also identify patients at risk of coronary obstruction. It provides additional information that is not available from MSCT or transoesphageal echocardiography (TEE) and can help predict how situations such as severe, eccentric calcification may behave and the complications that can arise from it.

Commonly in BAV disease the abnormal geometry at the annular level and unequal-sized leaflets makes alignment of the two or three hinge points (depending on BAV type) difficult. MSCT is useful in tricuspid valve disease to find the optimal implantation projection of the aortic root and an orthogonal alignment of the native annulus. This however, is often found to be unhelpful in BAV disease. Techniques have been described using the pigtail catheter and altering the fluoroscopic projection to find an optimal view for implantation; “follow the right cusp rule” (72) and the “Right cusp rule, Part II” (73).

### Valve crossing

**Figure 4:**
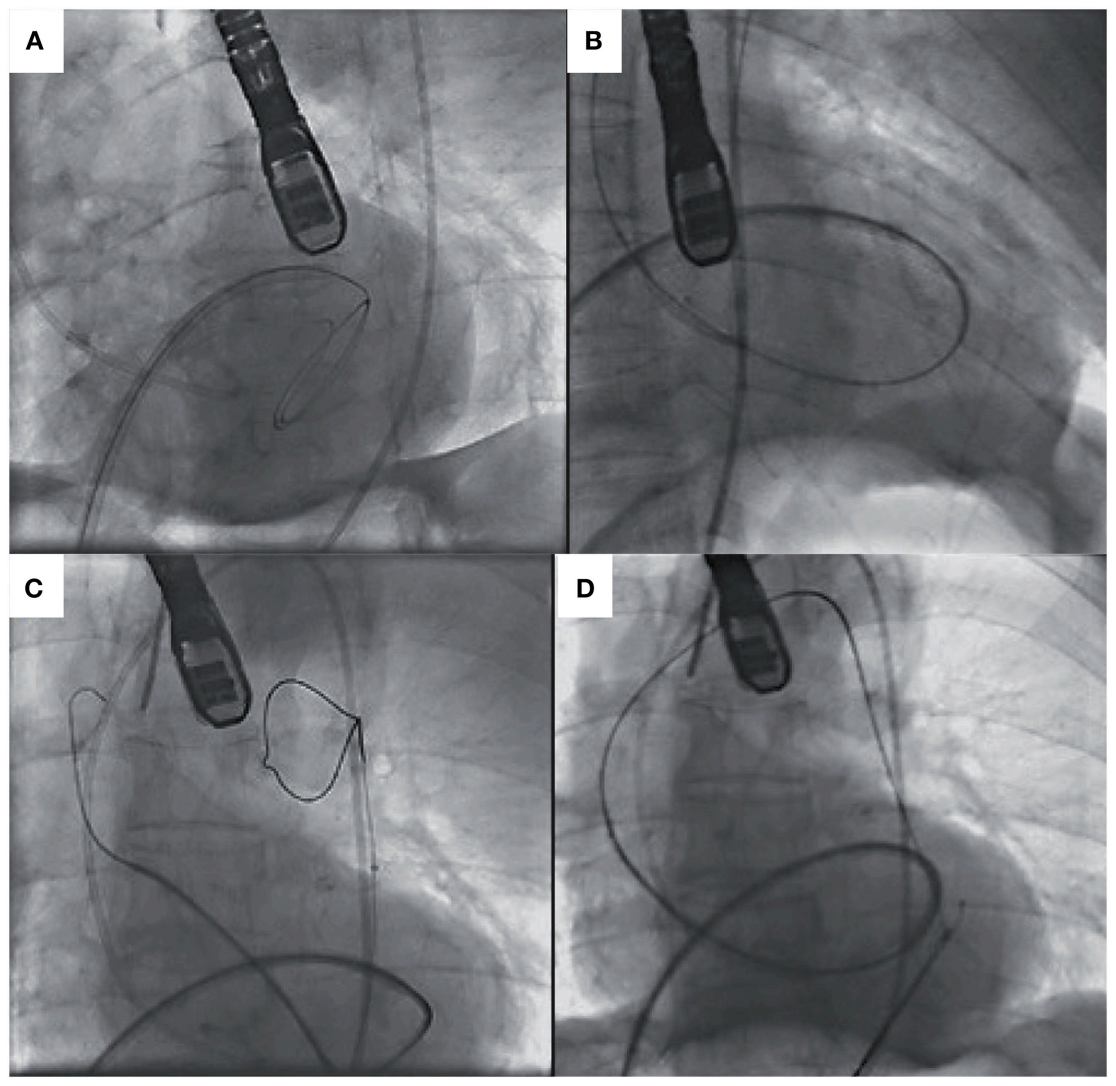
**(A)** Transeptal puncture and positioning of hydrophilic wire and Judkins Right in the left ventricle. **(B)** Anterograde crossing of the aortic valve and wire changed for exchange wire. **(C)** Amplatz Goose Neck Snare used to snare the exchange wire. **(D)** Exchange wire then externalized via the left femoral artery [Adapted with permission from the Rodríguez-Olivares et al. (75)].

Crossing the stenotic BAV may be challenging and time-consuming, increasing the risk of embolic cerebral complications. Careful interrogation of the MSCT can identify the fused cusps in BAV disease and may help with predicting the location of valve opening and maximize the chance of the wire to cross. Frangieh et al. (68) have described a step by step approach to crossing a stenotic BAV. Wire movement should start from the non-fused leaflet (“single cusp”) which has no raphe and then with careful rotation direct the wire in small steps toward the fused cusps. If starting in the NCC rotate clockwise, or anticlockwise if starting in the LCC. In BAV with no raphe, there are only two anatomical cusps and the guide wire should be slowly manipulated between each cusp carefully interrogating the opening between the leaflets. In more angulated aortic roots, catheters with a bigger curve such as an Amplatz-2 (AL-2) may help with retrograde crossing, and/or softer hydrophilic coated wires such as the Glidewire. In extreme circumstances when conventional retrograde wire crossing of the BAV is not possible, *ad hoc* trans-septal puncture followed by wire delivery into the left ventricular apex, anterograde aortic valve crossing, wire externalization and subsequent aorto-venous loop creation can be undertaken. A catheter of choice (i.e., AL) can then be placed retrogradely across the stenotic BAV thus facilitating regular fully percutaneous transfemoral TAVI (Figure 4) (74, 75).

## How to perform TAVI in bicuspid aortic valve disease

Historically, patients with BAV disease with severe AS were referred for SAVR. However, there is an increasing tendency for some younger patients to opt for a less invasive percutaneous procedure, particularly in the presence of significant co-morbidity or prior cardiac surgery. The selection of the type and size of the valve can be challenging due to the anatomical reasons outlined above, with MSCT playing an important role in THV device selection and implantation technique. Sizing and implantation technique is key to success in TAVI in bicuspid valve disease. Due the asymmetric nature of the aortic annulus, eccentric heavy calcification and raphe resistant to dilation, THV valves are implanted higher and anchored at the tightest part of the commissural. This has in part led to sizing of valves at +4 mm above the annulus at the intercommisural space. The final implantation depth are often higher due to the anchoring effect of the calcification at the level of the commisures with < 4 m for CoreValve, < 3 mm for CoreValve Evolute R (Medtronic, Minneapolis, Minnesota) and atrio-ventricular ratio of 60/40 for Sapien XT/S3 (Edwards Lifesciences, Minnesota). Here we present 2 illustrative cases of how to perform TAVI in BAV.

### Case 1

A 67-year old female with acute respiratory failure due to severe AS was referred for consideration of TAVI. She underwent ventricular septal defect repair at the age of 10. At the age of 57 she presented with progressive breathlessness secondary to severe mitral regurgitation and underwent a mitral valve replacement with a 27 mm Carpentier Edwards Magna Valve. Six years later she developed further progressive dyspnoea and underwent an urgent redo mitral valve replacement for a stenotic prosthetic mitral valve. A 33 mm Carbomedics Valve was inserted into the mitral position. She has end-stage renal disease requiring haemodialysis.

**Figure 5:**
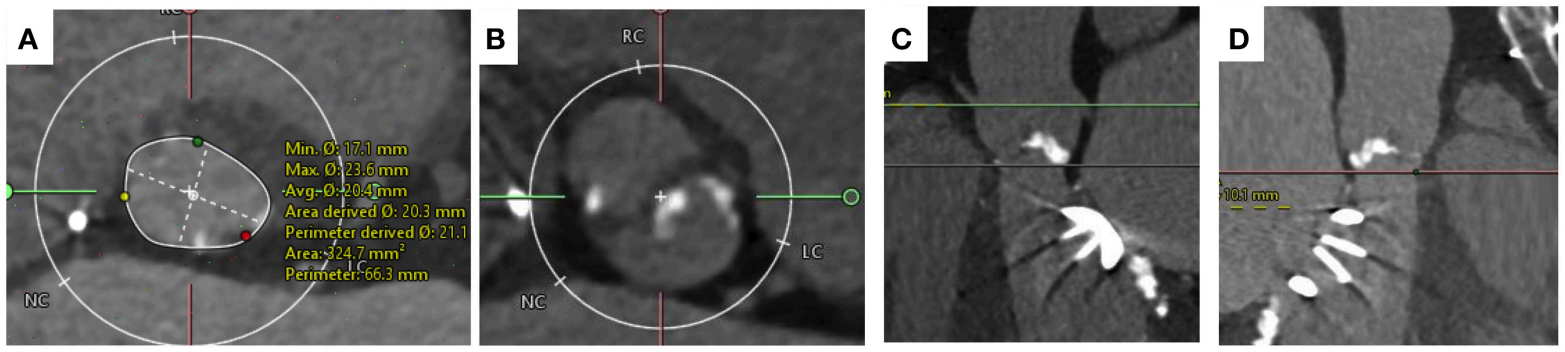
MSCT imaging of bicuspid aortic valve. **(A)** Annulus measurement with minimum diameter of 17 mm and maximum diameter of 24 mm and area of 325 mm^2^. **(B)** Cross-sectional view taken 4 mm above the level of the annulus. The valve is a Type 0 Sievers BAV with no raphe. **(C)** Distance to coronary ostia >10 mm. **(D)** The distance from the aortic annulus to the mitral valve ring measures 10.1 mm acceptable for TAVI implantation.

Transthoracic echocardiography showed a degenerated bicuspid aortic valve with a peak velocity of 5.2 m/s (peak gradient 108 mmHg; mean gradient 68 mmHg, area of 0.43 cm^2^). The mitral valve prosthesis was functioning normally. Invasive coronary angiography revealed normal coronary arteries. Following a Heart team discussion, it was decided to perform TAVI due to her previous mitral and redo mitral valve replacement. The aortic valve was a Sievers Type 0 bicuspid valve. The aortic annulus minimum diameter was 17 mm, maximum diameter 24 mm, perimeter 69 mm, and an area of 355 mm^2^ (Figure 5). The common femoral arteries measured 6 mm bilaterally. A technical concern regarding TAVI in this lady was the interaction of the TAVI valve with the mitral valve prosthesis and the risk of valve embolization during deployment. The MSCT identified the rim between the mitral valve prosthesis and the aortic annulus to measure 10 mm, which was felt to provide an adequate landing zone for the THV, with the risk of valve embolization deemed to be small.

**Figure 6:**
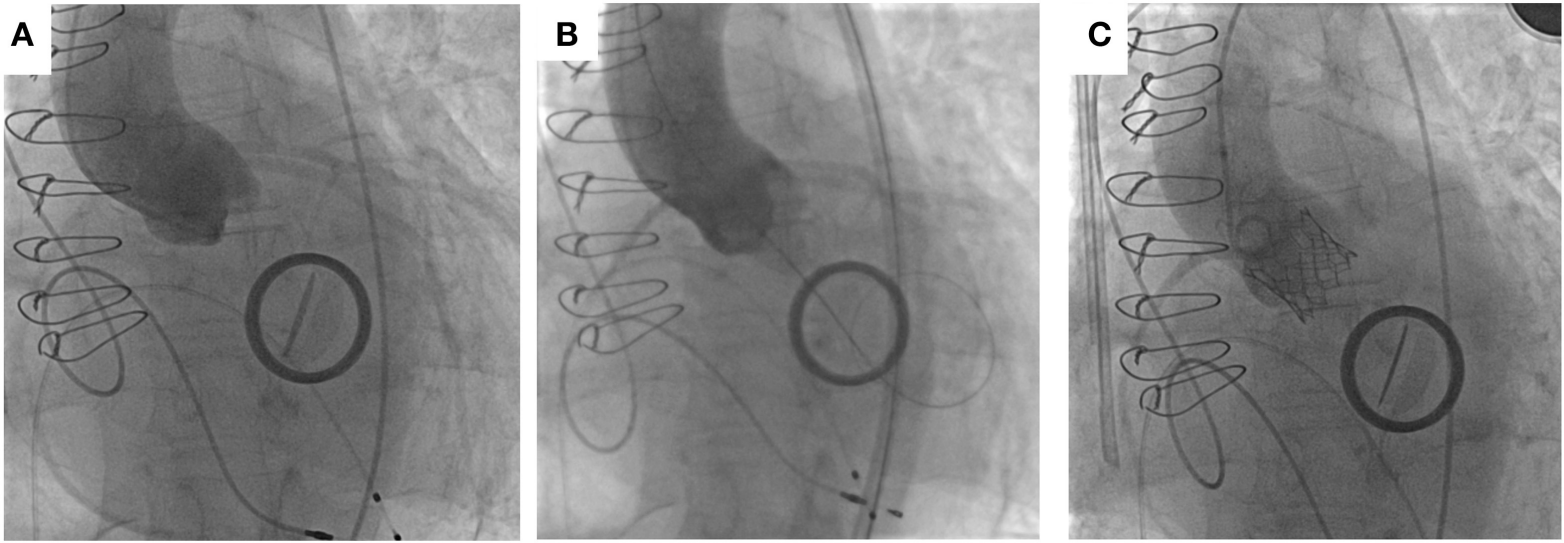
Implantation of SAPIEN 3 in patient with bicuspid aortic valve with previous mitral valve replacement. **(A)** Aortogram for root alignment with pigtail catheter in the right coronary cusp. **(B)** Balloon sizing with 20 mm balloon touching hinge points with simultaneous aortogram showing filling of coronary arteries. **(C)** Successful deployment of 23 mm SAPIEN 3 valve with no paravalvular leakage.

**Figure 7:**
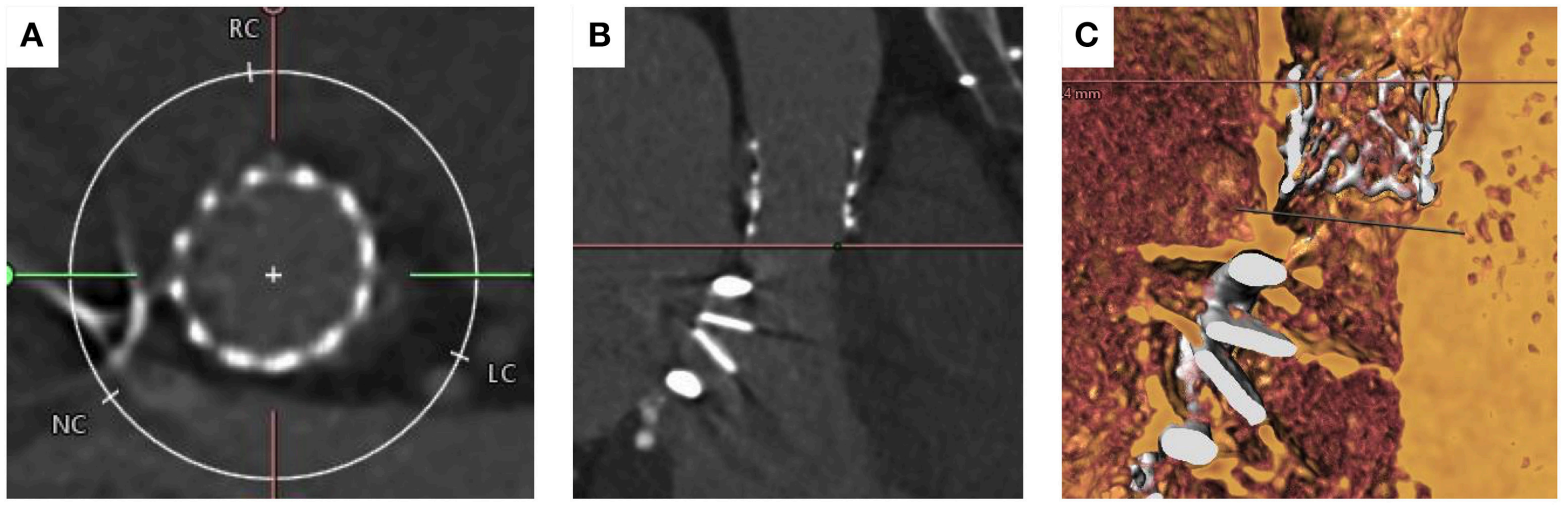
MSCT imaging of the aortic annulus post TAVI implantation with 23 mm Edwards Sapien Valve. **(A)** shows circular deployment of the transcatheter heart valve. **(B)** Left ventricular outflow tract view showing bi-leaflet mitral valve prosthesis and TAVI valve post deployment. **(C)** Volume rendered imaging confirming there is no interaction between the base of the TAVI valve and mitral valve prosthesis.

The right femoral artery was cannulated and a 14 French E-sheath was introduced. The annulus area was felt to be between two valve sizes (23and 26 mm Sapien S3 valves). The valve was crossed retrogradely using an Amplatz Left1 diagnostic catheter and a “straight” standard wire. The wire was then exchanged with a pre-shaped small Safari wire (Boston Scientific, Natick, MA, USA). A balloon aortic valvuloplasty was performed and simultaneous aortogram was performed. An aortogram confirmed that during inflation the 20 mm balloon adequately filled the aortic annulus and was in contact with the hinge points (Figure 6). Coronary filling was also visualized. It was therefore decided to implant a 23 mm Edwards Sapien S3 Valve. Careful attention was given during valve positioning ensuring the valve skirt was positioned just below the hinge points. The valve was confirmed to be well positioned with good hemodynamics without paravalvular regurgitation. Following valve deployment, MSCT was performed confirming circular expansion of the valve and no infringement on the mitral valve prosthesis (Figure 7).

In cases of BAV, large aortic annuli pose a challenge for the TAVI operator as large annular dimensions fall outside the recommended sizing ranges for currently available THVs. There are concerns regarding significant paravalvular leakage post TAVI deployment in this group of patients. In certain cases with large annuli the inter-commisural space can be used for the landing zone with good effect. Improvements in THV design (in particular the sealing skirt) and technical considerations such as landing the valve in the inter-commisural space means that these patients can be treated with good outcomes. This forms the rationale of some TAVI operators who suggest to size and land the THV 4 mm above the measured annulus.

### Case 2

**Figure 8:**
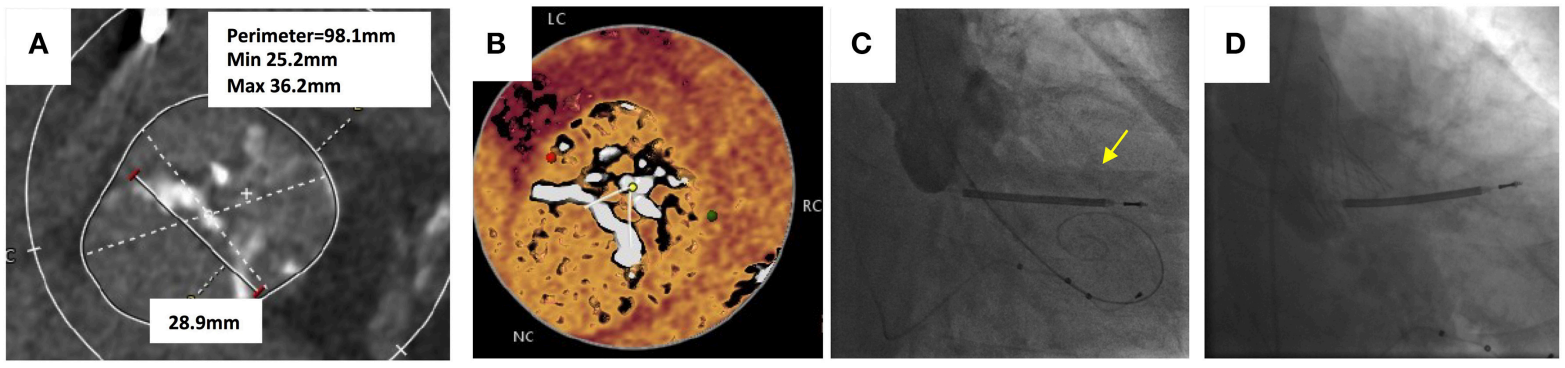
Transcatheter aortic valve replacement in bicuspid aortic valve. **(A)** Sievers Type 1 bicuspid valve with partial raphe between the right and left cusps. Large annulus with perimeter measuring 98.1 mm and an intercommisural distance of 28.9 mm. **(B)** “Hockey puck” view of the bicuspid valve shows partial raphe between left and cusps. **(C)** Balloon valvuloplasty with 25 mm balloon for sizing prior to TAVI implantation. Yellow arrow shows leak of contrast into left ventricle during simultaneous aortogram indicating inadequate seal. **(D)** 34 mm Evolute R deployed at leaflet tips with no paravalvular leak.

A 58 year-old male with end stage renal disease due to IgA nephropathy who had previously undergone a renal transplant which had failed was declined for redo renal transplantation. He had progressive dyspnoea and a history of syncope on exertion. The transthoracic echocardiogram confirmed severe stenosis of a BAV with a peak velocity of 3.9 m/s (peak gradient 61 mmHg, mean gradient of 39 mmHg and area of 0.89 cm^2^). There was also the presence of moderate aortic regurgitation. Coronary angiography revealed non-flow limiting coronary artery disease. MSCT confirmed a Sievers Type 1 bicuspid valve with a partial raphe between the right and left cusps. The aortic annulus was large with a perimeter measuring 98 mm, mean Sinus of Valsalva diameter of 40 mm and an inter-commisural distance of 29 mm (Figure 8). Both common femoral arteries were of large caliber and measured 8 mm on the left and 9 mm on the right. Following discussion by the Heart team and careful analysis of the MSCT it was felt that TAVI was technically possible if deployed at the inter-commisural space.

TAVI was performed under local anesthetic and the right femoral artery was cannulated and a 20 Fr sheath was inserted. The left femoral artery was cannulated and a 7 Fr sheath was inserted. The left femoral vein was used for insertion of a temporary pacemaker lead. The bicuspid aortic valve was crossed retrogradely using a Amplatz Left 1 diagnostic catheter and a hydrophilic coated straight tipped Glidewire. This was exchanged for a pre-shaped Safari wire and placed in the left ventricular apex. A balloon valvuloplasty was performed using a 25 mm balloon and a simultaneous aortogram was performed. The aortogram revealed a leak of contrast into the left ventricular cavity during balloon aortic valvuloplasty and felt to be an inadequate seal. A 34 mm Evolut CoreValve was deployed at the level of the leaflet tips, with hemodynamics and echocardiography confirming a good result with no significant PVL. The peak velocity across the TAVI valve was 1.8 m/s, peak gradient of 14 mmHg and area of 1.11 cm^2^.

## Conclusions

Despite encouraging data, especially with newer generation THVs for BAV disease, caution needs to be taken as patients with bicuspid AS are more likely to be younger (12, 38, 76) and therefore concerns regarding significant PVL and high permanent pacemaker implantation rates need to be addressed before offering it to younger and lower operative risk patients. With improvements in the design of the sealing skirts of THVs, PVL rates have reduced and the ability to reposition and retrieve the devices have led to more technical success with TAVI in selected patients with BAV disease. Procedural success is high and the survival rates are similar to those in patients with tricuspid valve AS undergoing TAVI. However, complications such as moderate or severe PVL and aortic root dissection are more common in BAV disease compared to tricuspid aortic valve patients. As the indications for TAVI expands with data supporting its use in the younger and intermediate risk group patients, the proportion of patients with BAV is expected rise. Specifically designed prospective studies are required to provide further evidence on durability, anatomical selection criteria and long-term success before it becomes a viable option for patients with BAV.

## Author contributions

RD and RP wrote the manuscript and provided substantial contribution to the discussion and content. All the authors researched data for the article, and reviewed and/or edited the manuscript before submission.

### Conflict of interest statement

The authors declare that the research was conducted in the absence of any commercial or financial relationships that could be construed as a potential conflict of interest.
